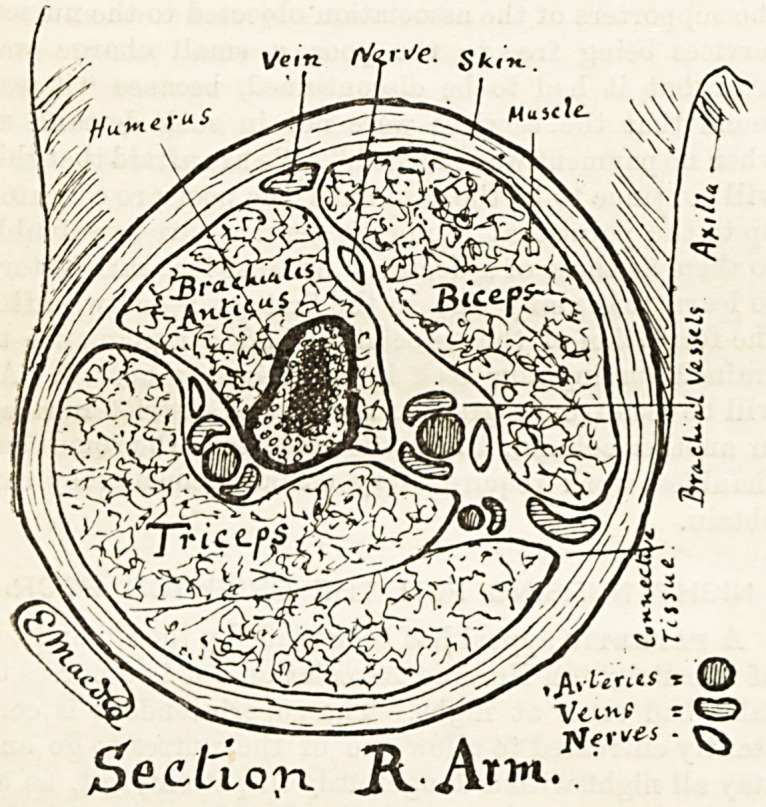# "The Hospital" Nursing Mirror

**Published:** 1900-03-17

**Authors:** 


### "The Hospital" Nursing Mirror.

Being the Nursing Section of "The Hospital."

[Contributions for this Section of "The Hospital" should be addressed to the Editor, The Hospital, 28 & 29, Southampton Street, Strand, London, W.C., and should have the word "Nursing" plainly written in left-hand top corner of the envelope.]

### Notes on News from the Nursing World.

### THE QUEEN AND THE NURSES OF ST. MARY'S HOSPITAL.

On Saturday, through the kindness of a lady at Court who is greatly interested in St. Mary's Hospital, the matron, four sisters, and six nurses were allowed to occupy places on the platform of Paddington Station to witness the Queen's departure from London. "Great," writes one of the chosen few, "was our delight. It was so nice to be expected and conducted to the spacious waiting-room with three large windows, which were placed at our disposal. Out in the crowd we espied a tiny child, who often comes to the out-patient department, armed with a large white handkerchief. The policeman who rode before the Queen proved to be none other than Mr. Blatchford, whose wife had undergone an operation for gall-stones a few years ago. When we heard the prolonged cheering and saw the waving of hundreds of handkerchiefs we knew what it all meant, and soon we saw our own brave Queen. Permission was then given us to go on to the platform, and some kind-hearted members of the Press made room for us, so that we got a splendid view. We saw the baby boy, Master Alexander Roberts, aged 18 months, present her Majesty with a bunch of flowers, and we also saw the Queen herself walking right into the train, assisted by her black attendant. We had no eyes for any of the other Royal ladies and gentlemen. After the train left the platform one of the authorities took us through the State waiting-rooms, where tea was laid for the Royal party. The cups were plain white and gold, with the Great Western stamp. In the street a casualty patient had the misfortune to forget her handkerchief; in sheer despair she held up her bandaged hand and waved it to and fro. She told me afterwards that that she felt quite sure the Queen understood her little difficulty, for she looked and bowed."

### THE QUEEN'S CHOCOLATE.

"The other morning," writes a Lancashire district nurse, "on entering a cottage to dress a man's foot which had been hurt in a coal pit, I found a woman waiting to show me something of great value wrapped up in a soft piece of paper. It was a piece of the 'Queen's chocolate' sent to her by her brother, a private, from the Modder River. I asked her what she was going to do with it, and she replied, 'If he comes back, nurse, I shall eat it. If he never comes back I shall keep it always.' Inquiring for the box, I was informed that it also had been sent home, had already been framed, and that it made a 'lovely picture.'"

### MORE NURSES FOR SOUTH AFRICA.

The under-mentioned nurses, all members of the Army Nursing Service Reserve, left for South Africa in the "Avoca" on Monday: Misses L. Ainsworth, N. V. Blythe, F. L. Carey, J. E. Church, S. Clark, L. M. Green, R. A. Humphrey, A. M. Joscelyne, M. W. B. Kendall, M. Lippiatt, M. T. MacAdam, M. McLeod, M. Pedler, A. J. Richardson, L. Shepherd, E. C. Stuart-Jones, B. Turner, L. Warriner, F. M. Wilkinson, and A. B. Wohlmann.

### THE TRAINING OF SOME OF THE NURSES.

Miss Lily Ainsworth was trained at the Royal Hospital, Salford, and was subsequently staff nurse at the same institution. She was then attached to the St. Helena Home, St. John's Wood, for four years, and has since been engaged in private nursing. Miss Nora Valentine Blythe was trained at the General Hospital, Worcester, and has since been staff nurse at the Leeds City Fever Hospital and at the General Hospital, Birmingham. Miss A. J. Richardson was trained at the London Temperance Hospital, became staff nurse in September, 1893, and since 1894 has been sister of the same institution. Miss E. C. Stuart Jones is the matron of the St. John's Hospital for Diseases of the Skin; and the committee, who have appointed Miss A. Mills as temporary matron, have agreed to keep her place open for her on her return. Miss Alice Beatrice Wohlmann was trained at the Throat Hospital, Golden Square, and the Royal Free Hospital, Gray's Inn Road. We again regret our inability to supply as full information as we should like to do respecting the training and antecedents of some of the nurses despatched to South Africa, owing to the fact of their names not being entered in "Burdett's Official Nursing Directory."

### ENGLISH NURSES SPAT UPON BY BOER WOMEN.

A correspondent from Cape Town, who has had a chat with Miss Young, one of the last to leave Johannesburg, where she was matron of the hospital when the war broke out, sends us the following account of the experiences of Miss Young: "On the Sunday," Miss Young said, "we had not the faintest idea of leaving; indeed, on the Monday I was making preparations for nursing the wounded children, as well as at the hospital, when I heard a rumour that we should either be turned out, or given work under untrained Boer women. The latter report proved to be true, and subsequently I was informed that no medicine would be entrusted to my nurses for fear they should administer poison to their Dutch patients. They were in every respect to take subordinate positions. Under these circumstances I naturally sent in my resignation with that of my staff, and it was accepted on the Tuesday morning." Miss Young having told our correspondent about her journey to Cape Town, the latter inquired if she hoped to go back to Johannesburg at the expiration of the war. "Yes," she rejoined, "I have applied to be reinstated, and I am told that I am asking for the skin of the tiger before the animal is shot." Miss Young, in conclusion, mentioned the following incident: "Several English nurses were asked to prepare a small hospital, and later to nurse wounded Boers in an outlying district in the Transvaal. I was one of them. When all was in order and ready for the patients to come in the nurses were sent back after being disgracefully treated, and even spat upon by the Dutch women of the neighbourhood. People in England should know to what indignities we have been subjected by the Boers."

### THE AMERICAN HOSPITAL SHIP "MAINE."

In aid of the American Hospital Ship "Maine" Fund a unique and interesting concert will be given at the Crystal Palace on April 4th, under the patronage of the Queen and the Marchioness of Lorne, by the pupils and ex-pupils of the Royal Normal College and Academy of Music for the Blind. Dr. Campbell, the principal and founder of the Royal Normal College, is himself an American. With the exception of Madame Albani and the Crystal Palace Choir, who have kindly consented to assist, the performers, whether orchestral, instrumental, or vocal, are blind; and the mastery and power which many of them possess over the instruments which they have been taught to use is wonderful. This concert certainly deserves to be ranked among the many remarkable efforts made to provide for the requirements of the sick and wounded soldiers.

### A FIELD FOR NURSES IN WEST AUSTRALIA.

Commenting on the wishes expressed by some of the West Australian nurses to be selected for service at the seat of war, the correspondent of a Perth paper points out that there is at the present time a great need for additional nurses at the Government hospitals on the gold fields at Coolgardie and Kalgoorlie. Here, it is urged, there is many a brave man on his back who badly requires the care of a well-trained nurse. It may perhaps be worth the while of some of our unoccupied nurses in England to turn their attention to the field of operations in West Australia.

### THE NURSING STAFF AT ST. GEORGE'S INFIRMARY.

It appears that not only has great indignation been excited amongst the nurses at St. George's Infirmary, Fulham Road, by the new departure, to which we alluded last week, but that they are also still suffering from over-work. Thus the probationers have written to the Infirmary Committee stating that the duties they have to accomplish are too heavy; that the nursing staff is too small to cope with the amount of work that must be done since the convalescent patients are not allowed to help; that the strain and the worry is injurious to their (the probationers') health; and that they are all thinking seriously of going and forfeiting their certificates, rather than stay and ruin their health. This letter is signed by 48 nurses. Perhaps even more significant than this is a letter from the Matron to the Medical Superintendent, in which, after referring to the conditions that obtain through insufficient nurses, she says: "In No. 7 Block we had a landing in charge of a probationer, who had not been here a fortnight. She had to take charge of 13 helpless and dirty old women; three of these were apparently dying. There are also two who are quite fatuous, and who wander about; there are three others needing more or less attention, three quite helpless children—one of them blind—and other patients, making a total of 31. I think the task put before this probationer was overwhelming, and I know she could not do it." Finally, Dr. Webster, the medical superintendent, supports the views of the Matron and nurses. In the face of this conclusive demonstration of opinion, it is impossible to imagine that the Guardians of St. George's, Hanover Square, who must include some humane gentlemen, will allow the persons who maintain that the strength of the nursing staff is sufficient to rule the roast.

### CHANGES AT THE LONDON HOSPITAL.

The resignation of Miss Sturge, better known at the London Hospital as Sister Harrison, will be regretted by friends in many quarters. She resigns her post in consequence of ill-health. Perhaps the most striking testimony which could be paid to her popularity among the patients is that they seldom wish to leave the Harrison Ward. She was also greatly beloved by all her colleagues. The resignation of Sister Moreing is also announced, owing to need of change and rest after her long service at the hospital. The vacancy caused by Miss Sturge's departure has been filled up by the promotion of Staff Nurse Loweth, which has given general satisfaction.

### NEW TRAINING SCHOOL FOR NURSES.

The guardians of the parish of Paddington have recently made arrangements whereby a more complete system of training can be carried out than has been possible in the past. Since the infirmary was opened 14 years ago regular courses of lectures have been given by the medical superintendent, and class instruction by the matron, but owing to want of accommodation and other reasons probationers have not been taken. A house close by in Sutherland Avenue has now been rented and furnished, which will be for the use of the night nurses until a suitable nurses' home has been built. The nursing staff is to be increased by six probationers, to whom a three years' certificate of training will be granted, provided that they conform to the regulations and pass an examination conducted by an outside examiner. It is intended to commence the new system early in April, or as soon as suitable applications have been received.

### NURSING IN THE CANARY ISLES.

Several inquiries having reached us lately with regard to nursing in the Canary Isles, the following information communicated by a correspondent, who has been a resident for some years, may be of use to our readers: "I do not think there is much work for an independent nurse to do, and it would be foolish for any one who had to live on her earnings to go out to the Canary Isles unless to an appointment. Continuous work would be doubtful, and hotel life is expensive. No lodgings are obtainable. Each of the English hotels has a nurse of its own, but of course they make arrangements early in the season. The hotels pay the nurse a fixed salary, and take the fees. Sometimes there is scarcely anything to do; at another time a rush comes, and the nurse being single-handed is very hard worked. The nurses are often at liberty to take outside cases when their services are not required at the hotels. There is also a small British Seaman's Hospital with a matron and two nurses. These usually sign a three years' agreement."

### THE NORTHALLERTON NURSES.

The eighth annual report of the North Riding Rural Nursing Association, of which Miss G. Atkinson is lady superintendent, shows that the nursing fees have increased to the extent of £154, and proves how largely the revenue is augmented by the additional number of nurses who have been placed upon the staff. In the rules concerning probationers, who are now received by the Association when there are vacancies in the Northallerton Cottage Hospital, we notice that every probationer received into the association must go for three months for trial, and if satisfactory will at the conclusion of that time be expected to enter into an agreement in writing with the committee to serve the association for the term of one year as a probationer, and for a further term of three years in addition to the year of probation as a nurse. Probationers receive no wages. As nurses the wages for the first year is £16, for the second £18, and for the third £20. One pound per quarter of each year's wages is retained by the committee, and will be paid at the termination of the period of the agreement. The age considered desirable is from 22 to 30.

### ONE SUBSCRIBER PAYS THE SALARY OF A NURSE.

The fourteenth annual report of the Southport and District Nursing Society, which was adopted the other day, shows that the staff of nurses is now larger than it has ever been. This is owing to the generosity of a single individual, who early in the year increased his annual subscription to the amount equal to the salary of a nurse on condition that an extra nurse was engaged. As may be imagined, the condition was gladly complied with; and a great many other nursing associations must wish that they possessed members both willing and able to subscribe £60 a year for such an admirable purpose. According to the Mayor of Southport, there is no institution in the town of a more useful character than the nursing society, and it may be added that last year the nurses paid nearly 17,500 visits.

### INCREASE OF THE NURSING STAFF AT WARRINGTON INFIRMARY.

In view of the opening of the new infirmary at Warrington, the Board of Guardians have decided to engage six additional assistant nurses. The staff will then consist of a superintendent, and at least 12 nurses, which, it is calculated, will be sufficient for the requirements of the institution. The Guardians, very wisely recognising the difficulty of securing the services of competent nurses, are advertising for them in good time in order that they may be ready to take up their duties as soon as the new building is opened. We observe, however, with regret, that the Guardians have declined to increase their annual subscription to the Warrington Nursing Institution, which is doing excellent work in the town, and is badly in need of further funds.

### A NURSES' RECEPTION.

The directors of Bovril (Limited) invite the attendance of members of the nursing profession at a nurses' reception on the occasion of the opening of the company's handsome new premises, 152-166, Old Street, City Road, on Wednesday, March 28th, at half-past three p.m. The factories will be inspected, and samples of the Government rations, which are being prepared for the troops in South Africa, will be shown. Cards of admission will be sent on application, and afternoon tea will be provided.

### DISTRICT NURSING IN CARNARVONSHIRE.

In the statement of the president of the Carnarvon District Nursing Association, which was adopted at the annual meeting, it was mentioned that as some of the supporters of the association objected to the nurses' services being free to the poor, a small charge was tried, but it had to be discontinued, because "it was found that the services were not in such demand as when no payment was charged." We are afraid that this will continue to be the case until the poor are educated up to the point that a nurse's services are as valuable to them as those of a doctor. At least it is satisfactory to learn that many who in the first year or two after the formation of the association had a repugnance to trained nursing now seek it of their own accord. As will be seen from a graphic account of district nursing in another column, those who undertake the somewhat thankless work require every encouragement they can obtain.

### NIGHT NURSING FOR THE BRIGHTON POOR.

A pathetic appeal has been made by the Committee of the Brighton Nursing Association for two nurses to take bad cases at night. The superintendent is constantly entreated to allow one of the nurses to go and stay all night with a dangerously ill patient, but, as at present constituted, the staff is not large enough for her to respond to such requests. It has therefore been decided to start a special fund with the view of meeting this most pressing want. The sum required for the salary and maintenance of a single nurse is £70 per annum, but there must be plenty of well-to do people in Brighton who are willing to subscribe £140 in order that the Nursing Association, which has done admirable work during the three years of its existence, may possess a staff sufficient to minister to the needs of the poor.

### SHORT ITEMS.

Last week a deputation from the Matrons' Council of Great Britain and Ireland, consisting of Miss Isla Stewart, matron of St. Bartholomew's Hospital, Miss M. Mollett, matron of the Royal South Hants Hospital, Miss Breay, and Mrs. Bedford Fenwick, was received by the Civil Lord of the Admiralty, who promised to bring before his colleagues the proposal to organise a naval nursing service.—The secretary of the Westbourne Park Institute has afforded much gratification to some of the nurses belonging to St. Mary's Hospital, first by making their tickets to the popular lectures at Westbourne Park Chapel transferable, and, secondly, by reserving places for them. However great the crowd may be, "the nurses' seat" is always zealously guarded.—At a meeting of the Edinburgh Town Council on Tuesday the Lord Provost intimated that he had received £100 from Miss Florence Nightingale towards the fund he is raising for a Scottish hospital for the troops in South Africa. Miss Nightingale, who spoke in high terms of the Lord Provost's proposal, said that Edinburgh was second to none in her medical institutions, and her Royal Infirmary was second to no other.

### Lectures on Nursing for Probationers.

By E. MacDowel Cosgrave, M.D., &c., Lecturer to the Dublin Metropolitan Technical School for Nurses.

### PART I.—ANATOMY AND SURGERY.

### No. 1.—The Tissues.

The body is composed of a number of tissues. These can be most conveniently studied in the solid parts, such as limbs, as in the organs in the cavities of the body these tissues are more complicated.

Section R. Arm.

Take a section across the arm between the shoulder and the elbow, and see what tissues appear. Inside is the bone, with its large marrow cavity ; then come three large masses of flesh —the muscles (these are separated by connective tissue in which run blood-vessels), arteries and veins, and nerves. Outside of all is the skin, which is elastic, and, being fitted on tightly, keeps all the parts compactly together.

The bone, as seen in the skeleton, is only the lifeless portion, composed of lime salts. If examined under a microscope it is seen to contain numerous openings and passages, forming a regular network. These, in the fresh state, are filled by living tissues supplied with blood-vessels and nerves. The dry skeleton weighs about two-thirds of the living bone ; the other third can be discovered by soaking a fresh bone in dilute hydrochloric acid until the lime is dissolved out, the part remaining has the shape of the bone but is soft and flexible.

Bone is covered by a strong fibrous material—the periosteum; this has the power of secreting lime salts and building them into bone, and so helps the development and regeneration of bone ; when a bone is broken the periosteum secretes the repairing material.

Ridges and rough places can be felt on bones; these give attachment to muscles, and the more muscular a person the better marked are these rough places.

Bones are sometimes immovably joined, as occurs in the head and in the face, the lower jaw being an exception.

When bones are joined with the power of movement they are generally expanded towards the ends which meet in the joint, and these ends are covered with cartilage or gristle, which is elastic and tough, so preventing jars and shocks, and not wearing out as bone would do.

Joints are of three classes : 1. Ball and socket, such as at the shoulder and hip. In this the head of the movable bone is rounded and works in a hollow formed in another bone. A ball and socket joint gives movement in every direction, the shallower the joint the more extensive the range of movement. 2. Hinge joint, such as the elbow and knee, and as seen in the nodding movement of the head. This only swings backwards and forwards in one direction, as a door does on its hinges. 3. Pivot joint, as in the elbow ; in this joint one bone works in a circular direction round another ; the pivot in the elbow enables the palm of the hand to be turned up or down ; another example between the two upper bones of the spine allows the head to be turned from side to side.

Joints are closed in by a thin membrane or capsule, which is in the form of a short, wide tube, embracing the ends of the two bones which meet in the joint ; this secretes a little synovial fluid, which is nearly as thick as white of egg, and serves to lubricate the surfaces.

Joints are strengthened by bands of strong, flexible, but inelastic fibres called ligaments ; these run here and there across the capsule in a slanting direction, strengthening yet not restraining movement. The muscles and their tendons also support joints, and help to keep the surfaces of the bones pressed together.

Bones are divided into four classes, according to their shape : (1) Long bones, such as those of the arm, forearm, palm, and fingers, and the corresponding bones of the lower limb ; each has a cavity in its shaft. (2) Short bones, such as those of the ankle and wrist ; they have a layer of hard bone outside, the rest being filled up with loose spongy bone. (3) Flat bones, such as the sternum or breast bone, the scapula or shoulder-blade, and the ribs. These have two hard surfaces with spongy bone between. (4) Irregular bones, such as those of the spine, the upper and lower jaw, and the other bones of the face.

Bones have three uses : (1) For support ; the other tissues being soft, the body could not preserve its shape except for the support of the bones. (2) For protection. The skull protects the brain, the spine protects the spinal cord, the chest bones protect the heart, lungs, liver, spleen, &c., and the pelvis protects the organs it contains. The large blood-vessels generally run close to bones and are protected by them. (3) As levers to increase the range of movement possible to the muscles.

The muscles seen in the section of the arm are for the purpose of movement ; when an impulse reaches them from the brain they contract, their ends coming closer together and the fleshy body swelling out and hardening. As the ends of the muscles are attached to bones, when they contract they must draw the bones closer together, the direction in which the freer bone moves being regulated by the formation of the joint. For example, the biceps—the large muscle of the front of the arm—is attached above the shoulder-joint and at the other end to the radius on the round rough prominence an inch or so below the elbow ; when the biceps contracts, its ends must come closer together, and as the shoulder cannot be pulled down the forearm must be pulled up. The biceps can only shorten itself about an inch and so can only draw up the part of the radius to which it is attached that distance ; but whilst the upper end of the radius works in the elbow joint the hand swings through part of a large circle, the one inch shortening of the biceps raising it about two feet.

Muscles may be attached directly to the bone by their fibres, but more usually the muscle tapers off into a strong fibrous tendon which is attached to the bone ; by this arrangement the numerous muscles of the fore-arm, for example, are able to act on the fingers without making the wrist unduly thick, or interfering with its free movement.

These muscles are called voluntary, as they obey the will and only act when so desired; like all other voluntary muscles they are red. Voluntary muscles are sometimes called skeletal, as they are attached to bone, to distinguish them from the involuntary or visceral muscles found in internal organs.

Involuntary muscles are pink; they act without requiring exercise of the will, so, whilst voluntary muscles are at rest during sleep, circulation, respiration, and digestion—all being due to involuntary muscles—go on just as well as when awake.

In the section of the arm two classes of blood-vessels can be seen, arteries and veins; the arteries and larger veins are close to the bone, other veins are near the surface just under the skin. Arteries carry the pure bright blood from the heart to the tissues; veins carry back the darker impure blood to the heart in order that it may be sent to the lungs to be purified.

It is when passing through the capillaries which connect the ends of the smallest arteries with the commencement of the smallest veins that the changes in the blood take place. The capillaries are so small that they can only be seen through a microsocpe; their walls are so thin that the nourishment and oxygen of arterial blood can pass out through them into the tissues, and the carbonic acid gas and worn-out tissues pass in to be handed on to the veins.

The nerves keep all the tissues and organs in touch, conveying the sensations of the different parts to the brain, and enabling the brain to direct and regulate movement. It is only the larger nerves that can be seen in the section, the fine branches that go to the tissues require a microscope.

The trunks of the blood-vessels and nerves run in the connective tissue. This tissue under a microscope looks like a quantity of white cotton cut into short lengths, and mixed up so that the threads run in different directions. These threads loosely attach the muscles, skin, &c., together, and give support to the vessels and nerves, but do not tie the muscles so tightly together as to interfere with their separate power of movement.

### Minor Appointments.

COLCHESTER HOSPITAL.—Miss Elizabeth Keith has been appointed Night Sister. She was trained at the Western Infirmary, Glasgow, and has since been engaged in private nursing.

SUFFOLK HOSPITAL, IPSWICH.—Miss Isabel Thompson has been appointed Sister. She was trained at Evelina Hospital for Children, London, and Addenbrooke's Hospital, Cambridge.

HOSPITAL ST. CROSS, RUGBY.—Miss J. E. Keyte has been appointed Staff Nurse. She was trained at the Royal Lancaster Infirmary, and has since been engaged as private nurse at the Sunderland Nursing Institution.

STANLEY HOSPITAL, LIVERPOOL.—Miss May Stainthorpe has been appointed Sister of the women's and children's ward. She was trained at the Middlesbrough Fever Hospital and at the Huddersfield Infirmary, where for the last six months' she has been staff nurse. The patients in the ward in which Miss Stainthorpe worked at Huddersfield have presented her with a clock and photograph frame.

ST. LEONARD'S INFIRMARY, SHOREDITCH.—Miss Grace Humphries, Miss Mary Shearea, and Miss Eva Maud Sheppard have been appointed Ward Sisters. Miss Humphries was trained at East Dulwich Infirmary. She has since been staff nurse in the same institution and sister of Stapleton Infirmary Bristol. Miss Shearea was trained at Mile End Infirmary, where she has since been staff nurse. Miss Sheppard was trained at St. Olave's Infirmary, Rotherhithe, and has since been engaged in private nursing.

### Nursing at Maritzburg.

BY AN ALEXANDRA NURSE.

Maritzburg, February 17th.

SINCE I wrote last three more of the soldiers' wives here have lost their husbands. The wife of one—a colour-sergeant in the Dublin Fusiliers—had only been confined of twins the previous week. Her husband was shot at Colenso, and although brought down to our camp here she was not able to see him, as he very soon passed into a state of unconsciousness. Since then she has been sent home with three delicate and fatherless children to care for, all girls, as the baby twin boy died after a very short existence. Another of the Dublin Fusiliers, also a sergeant, since dead, by his very anxiety to get back to his duties contributed in no little measure to his own illness. With the regiment he went to Dundee, contracted pneumonia, was laid up in Ladysmith for some time, then brought down here. After a time he left the hospital and resumed store duties. But he was struggling against health impaired, and typhoid followed, from which he never rallied. He was not really fit for work. One of his children at the same time was very seriously ill from fever and tuberculosis, and we were afraid there would be a double bereavement for the rather delicate and gentle wife. The little boy, however, is improving, and is able to run about these last two weeks. The poor mother suffers much from rheumatism, and is not very capable to battle with the world. Her great anxiety is to have her three children well educated, and I hope friends will be raised up to help her in this, the wish of her heart.

TYPHOID AMONG OFFICIALS.

In my last, you may remember, I spoke of our late dispenser as being now in charge of our hospital train, saying how well he was suited for this post from his genial and amiable manner. Well, to the regret of all here—for he also was a general favourite with the public—he was laid low from typhoid and died after a ten days' illness. His wife and four children had gone home in November and will sadly miss the husband and father. Disease, dysentery and enteric, are very busy in our midst. Sister Wilson (civilian nurse) is at present in Grey's Hospital suffering from enteric. She was in charge of the typhoid ward here for some time. Meantime the doctors speak favourably of her case, and we all pray for a speedy recovery. Having had enteric myself last year I can fully sympathise with her.

WORK AMONG THE OUT-PATIENTS.

Apart from the nursing of the women and children, 90 in number remaining, I attend the inspection room and dress the outdoor patients, those who leave hospital or who are not so severely wounded as to require indoor treatment. I usually have from 40 to 50 each morning, and find the work varied and interesting. So far these have made good recoveries and soon pass back to the front. The townspeople are very enthusiastic over our poor wounded, and gifts of all sorts are lavished upon them. One of the latest was a gift of deck and basket chairs to each of the hospitals, Fort Napier, the College, Legislative Assembly, and Grey's Hospital all participating. I know you are all remembering us at home, and I never go to an operation or see anyone seriously ill but my heart goes out to the absent wife, mother, or sister in the homeland. How I should like to whisper in her ear that loving hands minister to her dear boy!

### Wants and Workers.

WILL anyone give air cushion, pillow, or water bed for use in a large poor district? Air ring cushion is sorely needed. Any nursing appliances will be most gratefully accepted by Nurse Brocklehurst, 85, Thorpe Road, Melton Mowbray, Leicestershire.

### Nursing at the Chelsea Infirmary.

### A CHAT WITH THE MATRON.

By Our Commissioner.

The question of nursing at Poor Law Infirmaries is one of the utmost importance, and when I saw Miss de Pledge, the matron of Chelsea Infirmary, the other day, I had no difficulty in finding subjects for discussion. Naturally, however, the conversation first turned upon the nursing under her own auspices.

"Generally speaking," said Miss de Pledge, "I think the nursing at Chelsea Infirmary will compare favourably with that of any hospital. The nurses are, as a rule, refined and educated women, and I have every reason to be proud of them. Of course, there are great opportunities here for nursing—quite as great as in a general hospital."

"How large is your nursing staff, and what are the hours of duty?"

"There are thirty-six probationers and twelve charge nurses, one for each ward. The hours of duty for the day nurses are shorter than those in a general hospital. They start at seven a.m. and leave off at half-past seven p.m. The time allowed for dinner is three-quarters of an hour, for tea half an hour, and half an hour is also allowed in the morning for dressing. The latter is much appreciated. The night nurses go on at eight p.m. and are off at half-past seven a.m."

"And what about off duty?"

"The nurses are off duty four times alternate weeks, one of the periods being from five till ten; the other periods are from ten till one and two till five, so that, though the nurses do not get out every day, they have a longer period during the week. The other week they got from two to five, and one half day from two till ten. The charge nurses get a whole day once a month. As to holidays, the nurses have a fortnight in the year and the charge nurses three weeks."

"The holidays are not very long?"

"No; they are not, but we cannot have longer holidays unless we have more nurses. As it is, they begin in May and go on until November. Our off-duty times compare favourably with those of other infirmaries, and the holidays are equally long. There is a very comfortable Nurses' Home, and each nurse has a nice little bedroom to herself."

"How do your present regulations about duty compare with those of ten years ago?"

"The old system was to give leave from eight to ten—that is to say, when the nurses had finished their work. Our present regulations constitute one of the reforms we have been able to make. Infirmaries, as you know, started from a very low level, and are still tied hand and foot by the regulations of the Local Government Board."

"How many nurses are on duty in each ward?"

"Usually a charge nurse and a senior and junior probationer."

### The Bathing of Male Patients.

"I am going to ask you a question for which I must apologise, but, as you have perhaps noticed, in at least one provincial infirmary nurses have been called upon to bathe able-bodied male patients. What is your view of the point?"

"Such a thing, I am sure, was never contemplated, and it is entirely wrong to expect a nurse to be present in a bathroom when a male patient is bathed. In our wards we should ask a male convalescent patient whom we could trust to be present. The nurse turns the bath on, tests it, and then leaves the man in charge of the male patient. I should not tolerate the bathing of male patients here, and I do not think it would be tolerated in any London infirmary. It can never be necessary to put an able-bodied man into a bath. Nurses are always willing to wash a patient in bed, or, indeed, to do anything that ought to be done; but the bathing of male patients is outside that category."

### Medical Examinations.

"Supposing a nurse in your infirmary contracts an infectious disease, what happens?"

"The medical superintendent would see her, and, if he were satisfied that the disease was of an infectious character, she would be at once removed to one of the fever hospitals. Her friends are usually quite helpless, and we accept full responsibility in such cases."

"Is there any rule here under which the members of the staff have to submit themselves to a medical examination by any doctor whom the Guardians may appoint for that purpose, at any time they choose, the nature of the ailment being reported to them?"

"No, indeed, there is not, and I do not think any nurse ought to submit to such a rule. As to the idea of reporting the nature of an ailment to the Guardians publicly it is most objectionable. Some years ago there was a resolution here in force in reference to examinations, which, though not of the extremely offensive character you indicate, was keenly resented."

"How long ago was that?"

"The regulation was adopted September 26th, 1893, and rescinded in 1895. Although it was in operation for two years it was a regular farce the whole time, and was given up because even the Guardians who had previously supported it found that it would not work."

"Did it increase your difficulties in obtaining nurses?"

"Very considerably. In fact it became almost impossible to obtain suitable nurses."

### Dancing in Infirmaries.

"Would you like to say anything about dancing in workhouse infirmaries?"

"I do not approve of dancing in public institutions. By all means let nurses who wish go to private dances; but dancing where there are the sick and dying is altogether out of place. Nor do I think you will find that the better class of nurses would lend themselves to it. I believe that they take a higher view of their work. Dancing in such circumstances is not only incongruous, but it disorganises the institution for some time, both before and after the event."

### The Power of the Matron.

"Now about the power of the matron in a workhouse infirmary, Miss de Pledge?"

"She has just as much or just as little power as the medical superintendent feels inclined to concede to her."

"Which means, I conclude, that she is powerless?"

"Legally, yes. For instance, she cannot grant leave of absence for more than three hours to a nurse or a servant, or extend leave without the special sanction of the medical superintendent. The matron is still controlled by rules laid down for her guidance nearly thirty years ago, when there were no trained nurses in poor law infirmaries, and when the medical officer was the only person of any education in the building."

"What was the year in which those rules to which you refer were first enforced?"

"1872. Here is rule six, which still appears in the regulations for the matron: 'To be in the infirmary for the night by eleven p.m. unless she has previously arranged with the medical officer to return at a later hour.' The only duties apparently required by the Local Government Board from a matron are to see that the beds and bedding are kept in good order, that the cooking and other utensils are always clean, that the clothing for the use of the paupers is always neat, and that directions are given for the washing, drying, and getting up of the blankets."

"There is not much about nursing then, in the rules?"

"There is not one word about it. Since the rules were adopted, most of the infirmaries have become large training schools for nurses, and the rules ought, of course, to be entirely altered."

"Why should not the matron of an infirmary be in the same position as a matron of a hospital?"

"There is no reason whatever. Such a change would not make the matron regard the medical superintendent less as the head of the establishment, and by defining their respective positions would render it much more easy for them to work in harmony. Under existing circumstances it is no use to rail at a poor matron because she does not reform recognised abuses. The only thing she can do is to alter the hours of the nurses, and even that cannot legally be accomplished without the permission of the medical superintendent. The matron can often achieve something by persuasion, but my contention is that it should not be a question of persuasion, but a question of right."

"Meanwhile, you have made some improvements here?"

"Yes, and just recently. I was fortunate enough to obtain the approval of the guardians to a revised scheme for the training of nurses, and I am glad to say that since it came into operation last September it has worked exceedingly well. In the last ten years there have been enormous improvements here, chiefly in the *personnel* of the nursing staff, and I find that a very superior type of nurse offers herself now to the class upon whom we relied for a supply a decade ago. Since I have been at Chelsea Infirmary we have never had to advertise for probationers, and our best probationers become charge nurses, should there be a vacancy after their three years' training is completed."

### Private Nursing in India.

A brief account of a nurse's experience of private nursing in the North-West provinces of India may, perhaps, interest workers at home. A great field, no doubt, is open to English trained nurses almost all over India; indeed, they seem badly needed, but the difficulty of making a beginning, and of getting started on one's own account, keeps many nurses from travelling so far in search of work, who would otherwise venture.

### The Up-Country Nursing Association.

This reason led me to join the Up-Country Nursing Association as a means to an end, a means of knowing and being known; for when once well started, a trained nurse can earn quite twice the amount of pay working on her own account, especially if her training includes midwifery. The Up-Country Nursing Association has its headquarters at Naini Tal, a charming hill station. A picturesque little cottage, called Pilgrim Cottage, furnished and supplied with a staff of native servants, is provided for the nurses' use. They make this their home while they are waiting for cases. There are five nurses, but of course we were seldom or never all at the home together. In fact, the cottage is empty for months in the busiest part of the year—the end of the rainy season. We only nursed Europeans in their own homes, and we probably saw more cases of enteric fever than of any other disease.

### Nursing in the Hills.

After a short case in the plains in the middle of May, I travelled up for the first time to Naini Tal by the night train. Bareilly lies some 60 or 70 miles from the foot of the hills, and it seems absurd in England to say that we took from 11 p.m. one day to 5 a.m. next morning going that distance by rail; but I suppose the lines being laid mostly through jungle accounts for the slow progress. It was frightfully hot all night, and I shall not forget the heavenly freshness of the breeze, nor the beauty of the scenery, as we drove up the 22 miles of mountain road which lead to Naini Tal. We drove in a "Iongo," a rough two-wheeled cart, driven by an old grey-bearded native, looking very picturesque in his striped turban and horn slung over his shoulder. If I had been able to speak the language well enough to chat with him, I am sure he would have been a most interesting companion. My luggage was always a terrible anxiety to me travelling among the hills. On arriving at the station I was met by a chattering, gesticulating crowd of hillmen (coolies), wild-looking creatures clad in a mere apology for clothing; they gathered round my boxes and fought over them. At last, after no end of bargaining for pice (pence), the boxes were heaved on to the selected men's heads, and away they went with them, disappearing into the depth of the mountains. I wondered, with a silent prayer, whether my earthly goods would ever be safely returned to me. About 12 miles from Naini Tal we came upon a sweet little rest house (since partially destroyed by landslip). Here we breakfasted. The remainder of the journey was made by "dandy," a conveyance which rather resembles the old-fashioned sedan chair. It is slung on poles and carried on the shoulders of four coolies; rather a comfortable way of getting up the steep and narrow paths. At first I used to feel dreadfully sorry for the men carrying me, until I discovered how strong they were, and how well accustomed to carrying immensely heavy weights, principally on their heads. The scenery all the way along was perfect, so green and fresh with lovely ferns and moss and trees, reminding one of English lanes. Along the wayside we frequently passed groups of grey monkeys, and droves of camels with their wild-looking drivers, all adding to the charm and novelty of the scene. When at last I arrived at the Nurses' Home I found it shut up and deserted by the servants, who had gone off on their own account to smoke or sleep. After much shouting I roused the ayah and old khansamah (head servant), and got quickly settled in my new quarters. The next evening another nurse arrived from her case. She had been a U.C. nurse for four years, and was eagerly looking forward to her term of service (five years) being ended and her return to England.

### Nursing in the Plains.

I was soon recalled to the plains to a patient, but even that short stay in the beautiful climate had set me up, and I felt ready for any exertion. My patient was the wife of an officer. I found her in rather a miserable state, having arrived from another station a few days previously, and feeling too ill to superintend the unpacking or arranging of any things, so everything was in a muddle. The room which she was lying in was bare of all but a bed, table, and a chair; packing cases were piled up in the verandah. It was rather hard work nursing an enteric case in that temperature, as I had to take both day and night duty as well as I could. Luckily, the little ayah could carry out simple orders, and was devoted to her mistress. I found her a great help. The heat was at its worst. Just at sunrise and after sunset we get something approaching a breeze. Quite early in the morning houses in the plains have to be shut up, doors, windows, and shutters carefully closed to keep out the glare and the hot wind. If a crack was left open we suffered for it. About half-past six p.m., when the sun is low, the house was opened, and kept open all night. Towards the end of June

—a very trying time—even the nights seemed breathless. At last, however, the longed-for rains broke, bringing relief to us all. The sound of it splashing down on the dry-baked earth was lovely. I longed to run out in the verandah and paddle about in it. My patient made a capital recovery and I left her in July to go back to the home in Naini Tal.

### At a Small Hill Station.

My next journey was to a small hill station, about 37 miles from Naini Tal. The way lay through the mountains—narrow paths, with a steep, rocky hill on one hand and a deep "Kud" or precipice on the other. I was carried along in a dandy. We set out quite a cavalcade, as I had six coolies for the dandy and three for my luggage. We had some very nasty places to pass—gaps in the pathway which had been washed away in the rains. However, I placed implicit confidence in my men, who were as sure-footed as goats, and they managed beautifully. I longed to have someone with me to whom I could talk and share the lovely scenery. It was glorious—peak upon peak of mountain, melting away in the distance into a blue, like the sea. The mists sometimes quite obscured everything except a few feet of pathway ahead of us; and then suddenly it cleared, and showed the sun shining brilliantly, bringing out the most perfect lights and shades in the hillsides and valleys. I had to break my journey at a place called Peora; we reached it just after sunset. I have never experienced such an utter sense of loneliness as when my dandy was put down before the forsaken-looking bungalow, or rest-house. I realised I was entirely alone (except for a few natives) in the depth of the Himalayas. Mountains solemn, silent, and grand towered around and above me. Oh, how small I felt! However, after awhile the khansamah appeared, salaaming, and inquired what "My honour" would like for dinner. Lights were brought, and a cheery wood fire soon blazing. I made up my bedding before it, and was foon fast asleep. My coolies, who had passed the night in the verandah, woke me about daylight with their chattering. I was quickly dressed and ready to start, but was quite unprepared for the surprise I received on stepping out into the verandah. Right in front of me, rising up through the softest clouds, were the Snows! The early morning sun was shining on them, and they glistened like gold. How near they seemed, and yet how unapproachable, making one dream of "the Land that is very far off." I fancy the old khansamah thought me quite mad, standing there gazing speechlessly. Familiarity had bred contempt in him! To me it seemed worth going to India for alone. Unfortunately, I never reached Almorah, for as I was nearing it a note was given me by a servant, sent by the doctor, saying the child I was to have nursed had died during the night. So I retraced my steps to Naini Tal. Though that journey was in vain, I did not regret my experience of travelling in the Himalayas, and above all, the view of the Snows, which will always remain in my memory as "A joy for ever."

### Novelties for Nurses.

### A NEW MATERIAL FOR UNDERWEAR.

Dr. Thomalla's hygienic underclothing possesses many distinct advantages of its own. It is a manufacture of cotton and wool specially combined. The outside is of wool and cotton derived of their oils, whilst the inside is of raw cotton not derived of its oil. By this arrangement it is intended that the moistuie of the body shall pass through the inner side of the material, leaving it dry, and be absorbed by the outer material, thus avoiding the unpleasant clinging sensation peculiar to all-cotton or all-wool textures and minimising the risk of chill. The result is a well-woven elastic material, most agreeable to wear, and of good appearance. It does not shrink in washing and is durable. All manner of under-garments can be procured made of various thickness. The price is very moderate. The agents are Zimmerli and Handschin, 8, Love Lane, London, E.C., but purchasers must procure the clothing through their drapers.

### Nursing on the "Avoca."

### SOME BAD CASES.

A sister on board the "Avoca," which the other day brought 369 patients from South Africa—who are now in the Herbert Hospital, Woolwich—and returned on Monday, sends us the following note:

Our nursing staff consisted of seven civilian sisters and the acting superintendent, with fifteen R.A.M.C. orderlies, and some civilian attendants, who did the work of nursing remarkably well under the direction of the sisters.

### Five Deaths.

Unfortunately we had five deaths, but, considering the severe illness and weakness of so many of our patients, we were grateful that there were so few. Three died from dysentery, one from typhoid, and one from sunstroke. The case of typhoid came on board as dysentery. Another man, whose temperature had been 103 deg. 6, was doing remarkably well a few days before landing, temperature normal. Special precautions were taken that he should be treated as a lying-down case, and his cot was swung from the deck to the ambulance as it stood on the quay. The same course was adopted with all the serious cases.

### A Case of Paralysis.

One case of paralyis came on board at Durban from the "Lismore Castle." We all thought it impossible that he would reach England alive, but after many fluctuations he reached our shores. He was the last cot case to be removed. Brandy was given in ward and on deck, and the sister went to the ambulance with the doctor administering more before his final departure for the Herbert Hospital. The superintendent took special precautions that he should not be disturbed, and sent blankets, &c., from the ship in order to obviate any change until he got to his bed at Woolwich. I fear he is not long for this world, but his case is not hopeless because he rallied so frequently on the voyage.

### The Majority Convalescent.

Most of the invalids made such progress on the voyage that when they arrived in England they appeared almost ready for the front. We were most careful to give the men extra warm clothing as we came into colder latitudes. For this purpose we spent our spare time on the voyage in making chest protectors for the weakly ones. The Government gave fifty yards of flannel, and our thanks are due to the Women's Patriotic League, Durban, the National Aid Society, the Red Cross Society, and the A.M.B. Fund, who provided warm vests, pants, pyjama suits, overcoats, caps, mufflers, &c. In fact, it is difficult to say what they did not provide, including pipes, cigarettes, tobacco, and books, which luxuries could not have been provided by Government. All the officers were most kind and attentive, doing far more than their duty required. Each man had 4 oz. of port wine before landing and a hot dinner, so that they did not need the coffee provided by the Absent-Minded Beggars' Fund, but the soldiers all gladly availed themselves of the free telegrams to friends.

### To Nurses.

We invite contributions from any of our readers, and shall be glad to pay for "Notes on News from the Nursing World," or for articles describing nursing experiences, or dealing with any nursing question from an original point of view. The minimum payment for contributions is 5s., but we welcome interesting contributions of a column, or a page, in length. It may be added that notices of entertainments, presentations, and deaths are not paid for, but, of course, we are always glad to receive them. All rejected manuscripts are returned in due course, and all payments for manuscripts used are made as early as possible at the beginning of each quarter.

### The Commissariat Department.

By Helen Todd, Matron of the National Sanatorium, Bournemouth.

### I.—MEAT: THE PERCENTAGE OF ITS WEIGHT LOST IN COOKING, &c.

It is an extraordinary fact that nowadays when so much is demanded from a candidate for the office of matron or lady superintendent for any hospital in the way of nursing qualifications, very little stress is laid upon her housekeeping knowledge. Many committees seem to consider that the mere fact of the would-be matron being a woman is sufficient evidence of her capability to direct and control the domestic and commissariat departments of the institution. I well remember running the gauntlet of a certain county hospital committee when every conceivable question was asked as to my nursing qualifications, teetotal and religious convictions, ability to lecture to probationers, and manage a steam laundry and staff of private nurses, but not one member of that committee, and it was a large one, thought fit to make a single inquiry as to my experience in catering and housekeeping, which, indeed, were to be the elected matron's chief duties.

### The Value of a Course of Housekeeping.

In some few of our large hospitals a course of "housekeeping," generally lasting about three to six months, has been arranged for nurses who wish to obtain this special qualification. Speaking from experience I can testify to the great value of the teaching thus given, but unfortunately only a very limited number of applicants can obtain this training, and the great majority of nurses leave their hospital without any knowledge at all of its domestic departments. A nurse is certainly taught in our training schools a certain amount about dietary and food stuffs. She can probably repeat by heart the diet scale of her hospital with the variations required under certain conditions, she knows what the diabetic patient must avoid, and how to feed the convalescent typhoid. All this is part of her nursing training, but in nine cases out of ten she will find when occasion arises that she is supremely ignorant of "quantities" when dealing with them in the bulk, and that making out her orders and giving out her stores is a very serious matter when she has never been taught what joints are the most economical, how many pounds of beef should feed a certain number of patients, or how much sugar should be weighed out for a week's consumption. In the great majority of our smaller hospitals there is no such functionary as a steward, and therefore all stores and orders pass through the matron's hands. Her training in this respect is, therefore, almost as important as in nursing, and it is, as I said, a most extraordinary thing that it should have been, and still is, almost entirely neglected.

### The Difficulties of Catering.

One of the great difficulties in catering for large numbers of persons, each of whom is to have an exact weight of the food prepared, lies in the fact that much of the actual weight of the raw article of food is lost in the process of cooking, and so upsets all calculations which do not take this factor into account. The following facts, which have been worked out after many experiments bearing on this very question, cannot fail, therefore, to be of interest to many. Let us first consider the question of meat, a quantity not to be neglected in an English hospital. I have before me three different diet tables—one, that of a large London general hospital; the second, a children's poor-law school infirmary; and the third belonging to a sanatorium for consumption. The first allows on the "full diet" scale: Men, 6 oz. meat "dressed," and women, 4 oz. "dressed" per diem; the second table allows children from seven to eleven years of age 2 oz. of "cooked meat without bone," and children from eleven to sixteen years of age 3 oz. per diem. The sanatorium scale allows 12 oz. of meat per head per diem.

### The Cost of Beef and Mutton Compared.

It is easy, of course, to order so many legs of mutton or pieces of beef, but we must remember that for the sick we cannot re-cook our meat, neither do we wish for much cold left over. Now by a series of careful calculations we have found the loss of weight in the cooking of beef to be about 30 per cent., and that of mutton to be 50 per cent. At the first glance we might think beef therefore to be a more economical form of meat than mutton, and this is fully proved if we take also the price into consideration. Hospital contracts naturally vary, but we may reckon 6d. per lb. a very usual price for legs of mutton, and $7\frac{1}{2}$d. per lb. for topsides of beef; allowing each person 8 oz. of meat we find 100 lb. of beef will cost us £3 2s. 6d. The loss in cooking will reduce our beef to 70 lb. or 1,120 oz., it will, therefore, feed 140 persons, each costing $5\frac{5}{14}$d.; 100 lb. of legs of mutton will cost £2 10s.; of this meat 50 lb. or 800 oz. will be available for food, thus feeding 100 persons at 6d. a head; beef, therefore, is $\frac{9}{14}$d. a head cheaper than mutton; and in dealing with large numbers the saving represented by small fractions is by no means to be despised.

### The Advantages of Mutton.

Mutton is, however, generally preferred in hospital dietary, as it is usually both more tender and more digestible. To give the exact figures, as a specimen of the tables from which these calculations are taken, I find that on a certain day 336 children were to be provided with 3 oz. of cooked mutton each; 84 lb., without the bones, which we will consider presently when dealing with some of the legs separately, were prepared. After cooking the meat was found to weigh 63 lb., having lost 21 lb., 8 of which were consumable in the form of gravy. The loss of weight on the meat therefore in the process of cooking was exactly 25 per cent. Now let us consider some of the individual legs of mutton; one, the smallest, weighed when raw, $6\frac{1}{2}$ lb. The bone when scraped weighed 1 lb. 2 oz., and the meat alone after roasting, 3 lb. 4 oz., having lost in that process 2 lb. 4 oz. The total meat which could be served to the children from this particular leg of mutton was exactly 50 per cent. of its weight when delivered by the butcher. In some cases the loss is not quite so great, *i.e.*, in dealing with very much larger joints; thus, to take another example, we find a leg which weighed 9 lb. 1 oz. to have only 1 lb. 6 oz. of bone in it, and in cooking the weight of the meat shrunk from 7 lb. 11 oz. to 5 lb. 10 oz., the loss here being only $38\frac{8}{29}$ per cent. This shows us that within practicable limits the larger the joint the more economical the housekeeping.

Turning from the mutton table to that on beef, we see that for 370 children (140 adults according to the 8 oz. scale) 98 lb. of beef, without bone, were required; this after cooking weighed 70 lb. (not counting 11 lb. of gravy), showing a loss of 28 lb. on the whole, or $28\frac{4}{7}$ per cent.

With a clever cook and a well-regulated oven the loss is curiously constant; the figures jotted down on different days are, in many cases, identical, not having the slightest variation. In providing for patients on the "Salisbury" diet it is found necessary to allow $\frac{1}{2}$ oz. for "waste" to every 4 oz. required. Foreign meat is now very generally used in hospitals and other public institutions, and the old prejudice against it is fast dying out. American beef and Australian or New Zealand mutton are found the most satisfactory; in these days of quick transit American meat is not frozen, but simply stored in cool chambers, and this has done much towards making it more palatable and tender.

### Echoes from the Outside World.

AN OPEN LETTER TO A HOSPITAL NURSE.

Never again can it be said that English people are cold and undemonstrative! I thought that loyalty rose to considerable heights in 1887 and 1897, but then there was so much to feed it that one hardly wondered. The reception accorded to the Queen on Thursday, Friday, and Saturday last week was quite another matter, for, with the exception of half a dozen mounted police to clear the way for her carriage, and a few Life Guardsmen or Royal Horse Guards for the sake of appearance, the Sovereign was quite unattended, and she simply drove, as any of her subjects might have driven, through the streets of London. But what a drive it was! Hundreds and hundreds of men, women, and children lined the streets not quiet sightseers, but cheering, waving, shouting mortals, who sang "God Save the Queen" or "Rule Britannia" at every odd moment, whose faces looked so bright that they might have just heard a splendid piece of good news, and whose enthusiasm was almost beyond their own control. I frequently broke into laughter myself. I could not help it, the people were so funny, and yet at the same time a big lump rose in my throat, for there was something very touching in the signs of loyal love and appreciation which were apparent on every side. Respectable middle-aged men so far forgot themselves as to throw their well-brushed high hats into the air, oblivious of the sixpennyworth of "shine" which would be required anon; women whose hair was grey and their figures distinctly matronly sported huge flags, waving them, and shouting like their schoolboy sons; and as for the children, of course they went mad. Even in the evening, when the Queen had gone home—though not to rest, for each night there was a dinner party at Buckingham Palace—the people evidently liked to feel that she was in their midst, for they assembled outside her gates in large numbers, singing, cheering, burning coloured fires, and carrying flags, and deemed themselves happy indeed when the blinds were drawn up and their honoured Sovereign appeared for a minute or two at the central window. Her Majesty must have been delighted that she had come for two days to show herself—I hear the idea was entirely her own—when she saw the intense pleasure she had given. Thousands who would have been too timid to face the dangers of a crowd at a public function, took the opportunity of thus seeing their Queen quietly; and the "Relief of Ladysmith" and "The Queen Among Her People" will be two of the events of the spring of 1900 not likely to be forgotten. The scenes witnessed in London will probably be repeated in Dublin if the visit to Ireland becomes an accomplished fact. It is timed to take place at the beginning of next month, when the verdant isle will be looking its freshest and best, and it is hoped by the Belfast people that they too may get a peep of the Royal visitor.

---

Bloemfontein, the pleasant and prosperous capital of the Free State, has been reached, and the unconquerable French is once more to the fore. The actual occupation of the town can, therefore, only be a matter of a very little time. Meanwhile, the fact that the brother of President Steyn has been captured may rather disturb the President himself, unless he feels that, in view of coming events, his relative may be more comfortable as the guest of the enemy than if he were fighting. Certainly neither President Steyn nor President Kruger can derive any satisfaction from the withering reply of Lord Salisbury to the so-called "proposals for peace." It needed the audacity of a Kruger to suggest that, after all the lavish expenditure o British life and British gold, we should listen to a demand on the part of the Transvaal Republic for an international status which it did not possess under either the Convention of 1881 or of 1884. If I were a schoolmistress I should give my girls Lord Salisbury's epitome of the events which led to the war to learn by heart. It puts the whole matter in a nutshell so cleverly. Equally terse was the warning of Lord Roberts to the Presidents as to the abuse of the white flag. Surely the words, "such breaches of the recognised usages of war and of the Geneva Convention are a disgrace to any civilised nation," should make even the treacherous Boer flinch a little. The worst anxiety left is on account of Mafeking. Even now the relief may have come, although we do not know it; but to read that so long ago as February 23rd typhoid, dysentery, and diphtheria were epidemic; that it was impossible to isolate the cases properly; that the sufferings of women and children were terrible; and that horse flesh and bread made from horse forage was "all the food left," makes one's heart ache with sympathy and apprehension for the brave little garrison.

---

I wish some of you could have been with me on Monday at Devonshire House. I went to see the Prince and Princess of Wales inspect the Imperial Yeomanry Field Hospital and Bearer Staff, and partly owing to the delightful spring weather, the afternoon was most enjoyable. We all assembled on the raised terrace at the back of the house, and although the trees were still wearing their sombre suits of winter black they formed a pretty tracery over a tall piece of statuary which could be seen in the background. On the large and beautiful lawn, which shone like emerald in the sunshine, the men in khaki were drawn up, and the Royal visitors passed all along their lines back and front, whilst the rest of the gaily-dressed company, many in garden party frocks, watched the proceedings. The Princess, who looked exceedingly well and as young as ever, was, of course, in black, but the soft chinchilla on her velvet jacket made a pretty frame for her face. She also carried a muff to match on a gold muff-chain. The Prince of Wales made a nice little speech to the men, who were in luck's way, for after the ceremony of inspection was over the Princess stood on the terrace and handed to each man, with the sweetest smile, a neat brown paper parcel (which matched their clothes) containing some warm under garments. I heard a young doctor observe that he wished he had been a private that he, too, might have received a "gift from his Princess's hands."

---

Apparently, the Duke of Orleans' scandal is not going to die out so quietly as the principal person concerned must hope. You will remember that, according to a French paper, a short time after the publication of some of the drawings in which the English were held up to insulting abuse, the Duke wrote and congratulated M. Willette, the artist, upon his pictures, adding several statements, by no means of a complimentary nature, respecting the nation which, ever since he has found it impossible to live in France, has offered him its hospitality. The idea that the young man should have dared to have said a word against the Queen, who has always befriended him in every way, afforded him many marks of Royal favour, and even sometimes asked him to be her guest, naturally makes all English men and women burn with indignation. The Duke has denied that he ever wrote the offensive letter, but, as it is said to have been seen by several people in Paris, the facts are not easy to reconcile. The young man is to be given another chance of substantiating his defence. The committee of the Bachelors' Club propose asking him whether he wrote the communication complained of, and unless he be able to absolutely disavow it his name will be struck off the list of members of the club.

### Everybody's Opinion.

[Correspondence on all subjects is invited, but we cannot in any way be responsible for the opinions expressed by our correspondents. No communication can be entertained if the name and address of the correspondent is not given, as a guarantee of good faith but not necessarily for publication, or unless one side of the paper only is written on.]

### PRIVATE NURSING.

"The Nurses' Well Wisher" writes: Many nurses prefer co-operation, and rightly so, for they receive the bulk of their earnings and get much more freedom. But, as a friend, I should like to warn nurses not to abuse this freedom by behaving unprofessionally. Several good cases have been lost because people have got to hear of fast nurses who smoke, and they seem to think "co-operation" includes this class of nurses. I trust that all who wish to promote the welfare of nurses will exclude such women, who have evidently mistaken their calling.

### DANCES IN HOSPITALS.

"One of the Flock" writes: May I be allowed space in your valuable paper to say that I quite agree with "Matron" and "Nurse Ethel." Dancing should not be allowed within our hospital walls for many reasons. Is it not hard enough for suffering creatures to bear with patience their afflictions without being disturbed at night by the gay sound of dance music playing for a lot of giddy men and women, who jig away forgetful of the suffering their merriment may cause their unfortunate patients? In some places during that merry dance the Angel of Death may enter the hospital door to carry away an immortal soul to its eternal home. Is it right to disturb that dying one by worldly music? I say, decidedly no. Apart from the bodily suffering of those poor creatures in our hospital wards, is it fair to remind them of their infirmities by playing dance music within their hearing, which must bring back to them memories of days gone by when they, too, could have danced to the sound of those familiar strains? The staff of our hospitals should consist of women whose lives are entirely devoted to the noble work of nursing God's sick and suffering ones. They should be willing to give up pleasures of the world. No nurse can do her duty to her patients and have her head full of the dance which is to take place in the evening. No doubt many will say it is unfair to expect a nurse to give up all pleasures when she enters her profession, but my answer to them would be, If you are of the world, live in the world and enjoy the pleasures of the world; do not enter a life of administration to the sick and dying. And therefore I pray for the day to come when dances within hospital walls will cease.

### "NURSES WHO ARE ILL."

"A Lover of the Profession" writes: What kind of patients do we make, I wonder? Ideal ones we should be, I suppose! Yet, why?—for, after all, nurses are not made of different stuff to other people; although I verily believe it is sometimes thought so. Being but human, we have our little seasons of "the blues," and even of irritability, just like other folk. A chum of mine who is just struggling through a severe attack of diphtheria tells me she has learnt many lessons during her weakness—lessons which shall be used for the comfort and joy of her future patients. She is almost an ideal nurse, if one may say so much about a fellow worker, yet she tells me she makes a wretched patient, and knows quite well she worries the night sister, in whose ward she is, by being so irritable and restless, but she is unable to help it. Perhaps—who knows?—this is one of the lessons she is learning!—just to be a little more patient than ever with the sick folk, who are often so restless because they are so weary, and possibly worn, and sad. "I had no idea before," she says, "how one feels little kindnesses and attentions when one is very ill." If nurses do not catch the murmured thanks we may always feel sure there is a big throb of gratitude right down in the heart somewhere. Might this be another of the lessons, I wonder?—to be perhaps more ready than before to do the little deeds of kindness, so nameless, yet so numberless, which it is a nurse's privilege to be able to administer. None of us would wish our fellow workers to be ill to learn such little lessons, but may we not take a hint?—and if it be the means of prompting any one of us to listen more kindly to the (perhaps oft-repeated) pet worries of our patients, and to do what in us lies to ease their troubled minds, then, surely, the weariness and pain of my sick chum will not have been borne in vain.

### "CRIBBING" NURSES.

"A Patient" writes: I am supposed to be having an afternoon nap, but instead of that I am watching nurse who sits reading The Hospital, and at last frowning so fiercely at some passage in it that I laugh outright and ask: "What's making you so angry, nurse?" "Why," replies nurse, raising an indignant face, "Here is another Competition Question, and very severe remarks on the papers sent in, and some nurses have been cribbing again. It is really too bad." "Oh! well," I said, "'all sorts and sizes,' you know." "Yes; I know that," replied Nurse Mary, "but it is very hard lines all the same. Nurses, like great folks, are subject to such a severe fire of criticism, and most people are so given to judge all the rest by those specimens they happen to come across, that one backslider can do a terrible amount of damage." "Oh, well." I rejoined, "rush into print, write and remonstrate with all those naughty backsliders, imploring them to be good for the honour of their womanhood, their profession, and their training schools, and tell them Longfellow's beautiful lines:—

> 'O, what a glory doth this world put on
> For him who, with a fervent heart, goes forth
> Under the bright and glorious sky, and looks
> On duties well performed and days well spent!
> For him the wind, ay, and the yellow leaves,
> Shall have a voice, and give him eloquent teachings,
> He shall so hear the solemn hymn that Death
> Has lifted up for all, that he shall go
> To his long resting-place without a tear.'"

### Examination Questions for Nurses.

The following rules must be carefully observed by all competitors:—

Rules.

The competition is open to all. Answers must not exceed 500 words, and be written on one side of the paper only. The pseudonym, as well as the proper name and address, must be written on the *same* paper and *not on a separate sheet*. Papers may be sent in for fifteen days only from the day of the publication of the question. Failure to comply with these rules will disqualify the candidate for competition. Prizes will be awarded for the two best answers. Papers to be sent to "The Editor," with "Examination" written in the left-hand corner of the envelope.

N.B.—The decision of the examiners is final, and no correspondence on the subject can be entertained.

In addition to two prizes, honourable mention cards will be awarded to those who have sent in exceptionally good papers.

### Appointments.

Victoria Cottage Hospital, Emsworth, Hampshire.—Miss Ellen A. Taylor has been appointed Matron. She was trained at the Taunton and Somerset Hospital.

St. Mary's Children's Hospital, Plaistow.—Miss T. A. Bodington has been appointed Matron. She was trained at Nottingham Children's Hospital and Charing Cross Hospital, and has since been sister in charge of the male surgical ward of the Charing Cross Hospital.

### For Reading to the Sick.

JESUS has many lovers of His Heavenly Kingdom, but few bearers of His cross. He has many desirous of consolation, but few of His tribulation. He finds plenty of companions of His table, but few of His abstinence. All wish to rejoice with Christ, but few wish to bear anything for His sake.—*Thomas à Kempis.*

From strength to strength go on,
Wrestle and fight and pray;
Tread all the powers of darkness down,
And win the well-fought day!
Then having all things done,
And all your conflicts past,
Ye may obtain through Christ alone
A crown of joy at last. —*Wesley.*

**Reading.**

Begin at once; before you venture away from this quiet moment, ask your King to take you wholly into His service, and place all the hours of this day quite simply at His disposal, and ask Him to make and keep you ready to do just exactly what He appoints. Never mind about to-morrow; one day at a time is enough. Try it to-day, and see if it is not a day of strange, almost curious peace, so sweet that you will be only too thankful, when to-morrow comes, to ask Him to take it also—till it will become a blessed habit to hold yourself simply and "wholly at Thy commandment for any manner of service." The "whatsoever" is not necessarily active work. It may be waiting (whether half an hour or half a lifetime), learning, suffering, sitting still. But shall we be less ready for these, if any of them are His appointments for to-day? Let us ask Him to prepare us for all that He is preparing for us.—*F. R. Havergal.*

Every day let us renew the consecration to God's service; every day let us, in His strength, pledge ourselves afresh to do His will, even in the veriest trifle, and to turn aside from anything that may displease Him. . . . He does not bid us bear the burdens of to-morrow, next week, or next year. Every day we are to come to Him in simple obedience and faith, asking help to keep us, and aid us through that day's work; and to-morrow, and to-morrow, and to-morrow, through years of long to-morrows, it will be but the same thing to do; leaving the future alone in God's hands, sure that He can care for it better than we. Blessed trust! that can thus confidingly say, "This hour is mine with its present duty; the next is God's, and when it comes, His presence will come with it."—*Anon.*

Of nothing may we be more sure than this; that, if we cannot sanctify our present lot, we could sanctify no other. Our heaven and our Almighty Father are there or nowhere. The obstructions of that lot are given for us to heave away by the concurrent touch of a holy spirit, and labour of strenuous will; its gloom, for us to tint with some celestial light; its mysteries are for our worship; its sorrows for our trust; its perils for our courage; its temptations for our faith. Soldiers of the cross, it is not for us, but for our Leader and our Lord, to choose the field; it is ours, taking the station which He assigns, to make it the field of truth and honour, though it be the field of death.—*J. Martineau.*

### OUR CONVALESCENT FUND.

THROUGH the generosity of the subscribers to this fund, a district nurse has just been afforded the rest and change she needed after recovery from scarlet fever. She writes most gratefully as follows: "As soon as I am a little better off, I hope to give a subscription to the Convalescent Fund, and shall try and get some help from my friends, for I think it only right to such a good cause."

### Notes and Queries.

THE Editor is always willing to answer in this column, without any fee, all reasonable questions, as soon as possible.

But the following rules must be carefully observed:—

1. Every communication must be accompanied by the name and address of the writer.

2. The question must always bear upon nursing, directly or indirectly.

If an answer is required by letter a fee of half-a-crown must be enclosed with the note containing the inquiry.

*Swollen Glands.*

(229) Can you tell me if any parts of England are more suitable than others for children who suffer from swollen glands?—*X. Y.*

Dry, bracing localities are desirable as a rule. Margate has a special reputation for such diseases, but the advice of a medical man understanding the individual cases ought to be taken upon so important a matter as the choice of a residence for delicate children.

*Maternity Training.*

(230) Would you kindly tell me if the training at the Plaistow Maternity and District Nurses' Home, or at the East End Mothers' Home, is good? and I have had two years training in a hospital for women and children, and want to know if that and the L.O.S. would qualify me as a district nurse?—*E. G. B.*

The training at both is good, and the combination you mention would probably qualify you for district work. Why not, however, write and ask the General Superintendent, Queen Victoria's Jubilee Nurses' Association? If you wish to be a district nurse, it is best to be a "Queen's" nurse.

*Nursing in the Canaries.*

(231) I shall be greatly obliged for any information concerning nursing appointments in the Canary Islands, and also where to apply to.—*A. L.*

A note on this subject appears in the news columns this week. Application as to hotel nursing might be made to the Santa Catalina Hotel Company, 1, Laurence Pountney Hill, E.C.; or for the Métropole Hotel, Las Palmas, to Messrs. Elder, Dempster, and Co., Liverpool.

*Hard Water.*

(232) Would you kindly advise me how to soften very hard drinking water without machinery?—*A. M.*

The local chemist usually sells chemical preparations adapted to soften the water of the neighbourhood. Anti-calcaire serves the purpose very well in London, and is cheap.

*Almshouses.*

(233) Can you please kindly say if there is any publication which gives addresses and particulars of almshouses where a lady with very small means, and unable to add to them, can be received?—*W. L.*

A short list of such institutions is to be found in the "Englishwoman's Year Book" (2s. 6d. Adam and Charles Black.)

*Probationer.*

(234) I have a daughter (22) who wishes to become a hospital nurse. Will you kindly tell me the best way to proceed in the matter? She thinks she would prefer to learn in a children's hospital if possible.—*F. S.*

Your best plan is to secure a copy of "The Nursing Profession: How and Where to Train," as in it you will find a full list of hospitals that train nurses as well as their terms of service. You will then be in a position to select your training school, and should apply to the matron. The book is published by the Scientific Press.

*Cerebral Lesion.*

(235) Would you kindly let me know of any homes or institutions where a man suffering from cerebral lesion could be received? Nominal payments. Not insane or imbecile.—*H. B.*

The National Hospital for the Paralysed and Epileptic, Queen's Square, Bloomsbury, W.C., or the Hospital for Epilepsy and Paralysis, 52, Portland Terrace, Regent's Park, N.W., admit suitable patients at nominal terms. Apply the Secretaries.

*Woman's Military Hospital.*

(236) I would be very much obliged if you will tell me where I could apply or put my name down as midwife in woman's military hospital. I am a sergeant's widow, and a certified midwife, Rotunda diploma.—*Widow.*

The Secretary, the Soldiers and Sailors' Family Association, could probably advise you.

*Young Probationer.*

(237) I am most anxious to become a probationer in a children's or general hospital. Will you please let me know where to apply at my age (17)?—*Ethel B.*

You must wait at least another year before any institution would accept you. Study cookery and housekeeping, and see replies to other young probationers.

*Standard Books of Reference.*

"The Nursing Profession: How and Where to Train." 2s. net.
"The Nurses' Dictionary of Medical Terms." 2s. 6d. net.
"Burdett's Series of Nursing Text-Books." 1s. each.
"A Handbook for Nurses." (Illustrated.) 5s.
"Nursing: Its Theory and Practice." New Edition. 3s. 6d.
"Helps in Sickness and to Health." Fifteenth Thousand. 5s.

All these are published by THE SCIENTIFIC PRESS, Ltd., and may be obtained through any bookseller or direct from the publishers, 28 & 29, Southampton Street, London, W.C.

## Figures and Tables

**Figure f1:**